# Factors Affecting Arbovirus Midgut Escape in Mosquitoes

**DOI:** 10.3390/pathogens12020220

**Published:** 2023-01-31

**Authors:** Alexis Carpenter, Rollie J. Clem

**Affiliations:** Division of Biology, Kansas State University, Manhattan, KS 66506, USA

**Keywords:** arbovirus, mosquito, midgut, RNAi, apoptosis, basal lamina, antiviral immunity

## Abstract

Arboviral diseases spread by mosquitoes cause significant morbidity and mortality throughout much of the world. The treatment and prevention of these diseases through medication and vaccination is often limited, which makes controlling arboviruses at the level of the vector ideal. One way to prevent the spread of an arbovirus would be to stop its vector from developing a disseminated infection, which is required for the virus to make its way to the saliva of the mosquito to be potentially transmitted to a new host. The midgut of the mosquito provides one such opportunity to stop an arbovirus in its tracks. It has been known for many years that in certain arbovirus–vector combinations, or under certain circumstances, an arbovirus can infect and replicate in the midgut but is unable to escape from the tissue to cause disseminated infection. This situation is known as a midgut escape barrier. If we better understand why this barrier occurs, it might aid in the development of more informed control strategies. In this review, we discuss how the midgut escape barrier contributes to virus–vector specificity and possible mechanisms that may allow this barrier to be overcome in successful virus–vector combinations. We also discuss several of the known factors that either increase or decrease the likelihood of midgut escape.

## 1. Introduction

Mosquito-borne arboviral diseases continue to threaten the health and safety of a significant portion of the human population. The past few decades have seen the re-emergence of a number of destructive arboviruses including Zika, yellow fever, dengue, and chikungunya [1,2,3,4]. The threat of these diseases is increasing, with several models predicting that climate change will allow the spread of disease vector species into regions which were previously unsuitable for their survival [5,6]. Despite this urgent threat, there is still much that is unknown about how arboviruses interact with their arthropod vectors. One particularly vexing mystery is why viruses are often specific to certain vectors. This is likely due to numerous genetic and environmental factors, which may be unique to each arbovirus–vector combination. Nonetheless, if we could begin to understand why one vector can efficiently transmit a virus from one host to the next and why another is unable to, we may discover effective strategies to halt the transmission of arboviral diseases at the level of the vector.

To illustrate the specificity of virus–vector interactions, consider that, in the United States, there are over 200 species of mosquitoes, but only about 12 of those are known to be important in disease transmission [7]. In natural settings, the reason why some arboviruses are not transmitted by certain mosquito species can be due, in part, to incompatibilities in geographical range or host-feeding preferences. However, this does not fully explain virus–vector specificity, as even when range and behavior align this does not guarantee the ability to transmit an arbovirus. Conversely, some mosquito species can be experimentally infected with arboviruses for which they are not known to be a natural vector, as demonstrated by successful laboratory infections of *Aedes aegypti* with Sindbis virus (SINV) [8]. The ability of a mosquito to transmit a particular virus, or its vector competence, has been shown to be determined by complex interactions between the genotypes of both the mosquito and virus [9]. Vector competence is not static; for example, a single mutation in the chikungunya virus (CHIKV) genome was found to improve the competence of *Aedes albopictus* for this virus [10,11]. This adaptation has driven increased outbreaks and the geographical spread of CHIKV [12].

**Figure 1:**
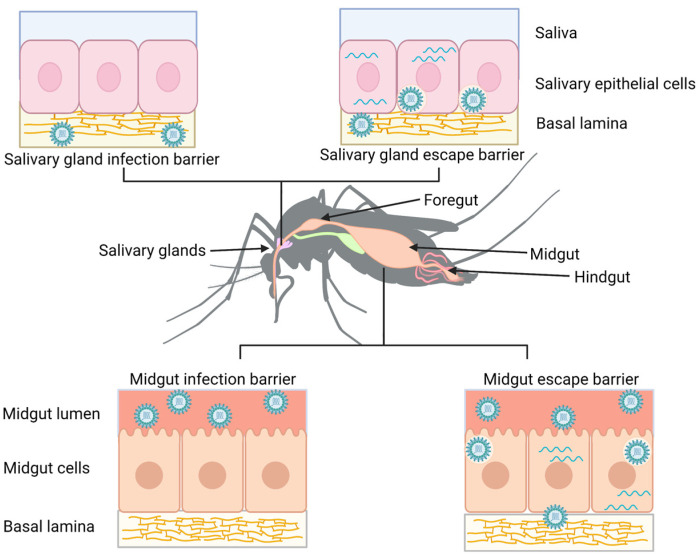
Tissue barriers to arbovirus infection. A midgut infection barrier (bottom left) occurs when viruses are unable to enter or unable to replicate in midgut epithelial cells. In contrast, a midgut escape barrier (bottom right) occurs when viruses are able to infect and replicate in midgut cells but do not disseminate from this tissue. A salivary gland infection barrier (upper left) occurs when the virus fails to infect the cells of the salivary gland, while a salivary gland escape barrier (upper right) occurs when the virus is unable to pass into the saliva. Figure created with Biorender.com.

There have been numerous factors that have been shown to influence the ability of a vector to transmit certain arboviruses (in this review, we will focus on horizontal transmission via hematophagy and ignore vertical transmission, which can also be important for certain arboviruses). One of the more well-studied factors is the presence of tissue barriers within the vector that the virus must overcome in order to be transmitted to a new host (reviewed in [13]). These barriers include the midgut infection barrier, midgut escape barrier, salivary gland infection barrier, and salivary gland escape barrier (Figure 1). A successful virus must overcome the midgut infection barrier by having the means to enter and replicate in the cells of the mosquito midgut epithelium after being ingested in a blood meal. It then must overcome the midgut escape barrier by exiting out of the midgut and infecting other mosquito tissues. Similarly, the virus must overcome the salivary gland barriers by infecting and escaping from salivary gland epithelial cells to be transmitted in the saliva the next time the mosquito takes a blood meal from a new host. If a virus is unable to overcome all these tissue barriers, then transmission will not occur.

A growing body of evidence suggests that the interactions of an arbovirus with the midgut of a mosquito have a significant effect on whether that virus will ultimately be able to be transmitted. While a midgut infection barrier can often be attributed to virus–receptor incompatibilities or immune responses in the midgut, the midgut escape barrier is more puzzling. A virus that encounters a midgut escape barrier is able to successfully infect and replicate in midgut epithelial cells, but it fails to reach any other tissues and is unable to be transmitted. This makes exploiting this barrier a promising means of arboviral control. However, our basic understanding of what constitutes the midgut escape barrier and how this barrier is overcome is woefully incomplete. The available evidence suggests there is no single factor that permits midgut escape but rather a constellation of factors that align in successful virus–vector combinations. In this review, we aim to discuss several of the known factors that affect midgut escape. We first discuss the significant obstacles that the midgut presents to a virus and consider some alternative hypotheses about how dissemination occurs. We then attempt to unravel the many external, viral and vector factors that seem to affect whether midgut escape occurs.

## 2. Routes of Midgut Escape

For a virus to escape from the midgut, it not only needs to overcome the midgut epithelium itself, but it also needs to pass through the dense fibrous extracellular matrix underlying the midgut called the basal lamina (Figure 1). The pores in the midgut basal lamina have been determined to be smaller than most arboviruses [14]. For this reason, there have been several proposals about how exactly a virus is able to overcome this obstacle that involve either location within the gut or mechanism of dissemination. In the former category are two major ideas: (1) dissemination occurs from the cardia/intussuscepted foregut; and (2) dissemination occurs from the posterior midgut. In addition, there are hypotheses about how the virus can cross the extracellular matrix/basal lamina: (1) virions can pass through gaps that are large enough to allow this directly; or (2) another tissue such as the tracheae penetrates this layer, allowing virions to escape the midgut without needing to navigate the basal lamina [13]. It is important to note that these ideas are not necessarily mutually exclusive, and it is also possible that there are different mechanisms of dissemination in different virus–vector combinations.

As the posterior midgut is the site of blood digestion in mosquitoes and most arboviruses are found to infect this tissue, it is generally thought to be the primary site of dissemination [15,16,17,18]. The idea that gaps or pores may exist in the basal lamina that are large enough for viruses to pass through was among the earliest hypotheses regarding midgut escape [14]. The distension of the mosquito midgut following blood meal ingestion leads to obvious questions about what this stretching does to the structure of the basal lamina. Despite this, clear-cut gaps in the basal lamina associated with escaping virions have not been definitively identified. However, an increasing body of evidence suggests that structural changes in the basal lamina may result in a possible escape route. For example, modified basal lamina which appears more porous or distorted and is associated with the visceral muscles has been reported after ingestion of virus-containing blood meals [19,20]. Even so, arbovirus escape from the midgut often takes days (depending on the type of virus), so structural changes would need to persist long enough to allow for this to happen. Supporting this idea, in a study that utilized gold-nanoparticles given in a blood meal, it was found that the mesh width of the basal lamina remained expanded even after the blood meal had been digested [21]. Some disruption of the basal lamina may be associated with cell degeneration as described by Weaver et al., who found that pathologic changes in the midgut occurred in Eastern equine encephalitis virus (EEEV)-infected *Culiseta melanura*, including some cellular degeneration which was associated with basal lamina disruption [22]. Passage through the basal lamina may lead to virus escaping directly into the hemocoel, which would allow the virus to access additional tissues, or the virus may need to infect another tissue such as the trachea before further spread occurs. While gaps in the basal lamina remains a viable and supported hypothesis of midgut escape, further studies will be needed to generate conclusive evidence.

More anterior regions of the digestive system have also been implicated as sites of dissemination. In this hypothesis, the cardia or intussuscepted foregut become infected and then the virus can cross into the hemocoel or into another tissue, such as the tracheae. The cardia is an organ that exists at the junction of the midgut and foregut, containing cells from both types of tissue, and is closely associated with tracheae and muscles. It is surrounded by modified basal lamina which appears more porous and thus may be more permissive for viral escape [23]. The idea that this region might be important for disseminated infection was derived from the observation that when *Culex pipiens* mosquitoes were fed with Rift Valley fever virus (RVFV), most of the mosquitoes that did not develop disseminated infection had virus detected in the midgut and did not have infection in the intussuscepted foregut, while most mosquitoes with disseminated infection had infected cells in this region [24]. Further studies were able to capture images of virions budding from cardial epithelial cells and virions in the basal labyrinth and matrix of these cells [23]. By studying the infection patterns in orally and thoracically infected mosquitoes, the authors of this study suggested that infection of the cardial cells would lead to spread of the virus to the intussuscepted foregut, and possibly to more anterior regions of the gut, before dissemination into the hemocoel by utilizing the larger gaps in the matrix [23]. However, this is not a likely route in all virus–vector combinations. For example, dengue virus-2 (DENV-2) was not consistently observed to infect the cardia in *Ae. aegypti* mosquitoes [18] and, while West Nile virus (WNV) antigen was detected in the cardia and intussuscepted foregut of *Culex pipiens quinquefasciatus* mosquitoes, this appearance occurred at approximately the same time as appearance in the salivary glands [16]. Thus, this anatomical location may play a critical role in dissemination of some viruses while playing little to no role in others.

Another means of viral escape from the midgut may be via the tracheal system, which extends throughout the body of the mosquito and provides for gas exchange. All insect tissues must be in close association with tracheal branches to survive, including the midgut. Evidence has shown that the tracheae may be a route of midgut escape for other types of insect viruses in other insects; notably, baculoviruses have been shown to use this route in lepidopteran larvae [25,26]. The evidence for arboviruses using this route in mosquitoes is not as conclusive; nonetheless, some studies such as those discussed below have indicated that this may be a viable hypothesis. Importantly, studies have shown that tracheae may penetrate into the midgut basal lamina in mosquitoes, providing the proximity needed to assist in midgut escape [19,27]. Infection of tracheal cells also appears to occur in a range of arboviruses such as DENV, Venezuelan equine encephalitis virus (VEEV) and RVFV [18,19,27]. Some of the most direct evidence for this hypothesis came from a study which concluded that, when injected into the hemocoel, VEEV needed to infect the trachea and muscles before midgut infection could occur [19]. A major caveat to this study is that it examined virus movement in the reverse direction, which may or may not reflect what happens naturally. In contrast, another study using CHIKV concluded that tracheal infection was not a necessary step in viral dissemination [20]. There is a possibility that the involvement of trachea in midgut escape is more relevant in certain virus–vector combinations such as those mentioned above, and its importance may be better understood with additional research.

## 3. Factors Affecting Midgut Escape

Each tissue barrier provides an obstacle to virus transmission, and learning more about these barriers may provide insights needed to combat arboviral disease. Regardless of the exact mechanism of midgut escape, the phenomenon of midgut escape barriers has been noted for decades across a wide swath of arboviruses and mosquito species (Table 1) [28,29,30,31,32]. Studying a midgut escape barrier is not straightforward, as its presence or absence is almost never absolute within a given population, and the percentage of mosquitoes exhibiting a midgut escape barrier can vary widely in different populations of a species in which some members are known to be able to transmit a particular virus. For example, one study that looked at the susceptibility of different *Ae. aegypti* populations in the United States and Mexico to DENV found that the percentage of mosquitoes with a midgut escape barrier varied from 4–43% [30]. Genetic and physical attributes of both a given arbovirus and mosquito species as well as environmental factors are among the many components that play into midgut escape. A review of some of the most important known factors are discussed in the following paragraphs.

### 3.1. External Factors

It has been well established that environmental factors can influence whether a virus is able to escape from the midgut of a mosquito. One of the most well-studied factors is temperature, but studies have also shown that other environmental components such as insecticide exposure and larval density may also play important roles.

- Temperature

The effect of temperature on midgut escape has been demonstrated in several different combinations of mosquito species and arboviruses. It has been found that when adult *Culex pipiens* mosquitoes were infected with WNV and held at higher temperatures (ranging from 28 °C to 30 °C), midgut escape happened faster, and ultimately more mosquitoes developed disseminated infection when compared with mosquitoes held at lower temperatures (ranging from 18 °C to 26 °C) [35,36]. This effect has been shown to extend to other combinations of vectors and viruses including in *Ae. albopictus* infected with DENV [37], *Ae. aegypti* with CHIKV [38], and *Culex* with St. Louis encephalitis virus (SLEV) [39]. Several reasons for these results have been suggested, including that higher temperature may increase viral replication within the midgut or may cause increased midgut permeability. The effect of temperature is complicated by genetic differences in mosquitoes and in viruses. Temperature has been shown to have less of an effect on midgut escape in some mosquito strains and some viral strains while having a greater effect on others [38,40].

In addition to the temperature at which adult mosquitoes are held, the temperature during larval development may ultimately affect viral midgut escape. One study investigating *Ae. albopictus* and CHIKV found that rearing larvae at lower temperatures was associated with increased rates of dissemination in adults [41]. Conversely, another study found that at low larval densities, increased temperature was found to increase the dissemination rate of SINV in adult *Ae. aegypti*; however, no difference was seen in dissemination when larval density was high [42]. The authors hypothesized that the larval temperature may alter adult mosquito immune gene expression such that mosquitoes reared at low temperatures were less competent vectors. These studies show that there is a need for more research that examines how vector competence is affected by different combinations of larval environmental factors.

- Exposure to pesticides

Another environmental factor that may affect midgut escape is exposure to chemical or biological pesticides. An unintended side effect of pesticides may be an increased dissemination rate in mosquitoes that are exposed at a sub-lethal level, as this has been shown in several studies. Bifenthrin has been shown to increase dissemination rates of Zika virus (ZIKV) in *Ae. albopictus*, with a particularly strong effect seen in older mosquitoes [43]. However, the same insecticide appears to have little effect on DENV dissemination [44]. Sub-lethal insecticide exposure may be particularly important for vector competence when larvae are exposed, as larval exposure to malathion has been shown to increase dissemination rates of SINV [45,46]. A possible reason for this may be that larval exposure to these insecticides results in adult midgut deformities, as has been shown to occur when mosquito larvae are exposed to the insecticide spinosad [47]. It has also been shown that sub-lethal exposure of larvae to the bacterial insecticide *Bacillus thuringiensis subsp. israelensis* caused increased rates of dissemination of DENV, although this was bacterial strain-specific [48]. It will be increasingly important to consider this possible side effect when treating disease vector-infested areas with insecticides.

- Larval density/competition

Other larval environmental conditions have also been shown to affect midgut escape, with some studies suggesting a link between larval density and viral dissemination rates in adults. In one study, when *Ae. albopictus* mosquito larvae were reared at higher densities, the adult mosquitoes had higher rates of disseminated infection; however, this effect was not seen in *Ae. aegypti* mosquitoes. It is unclear exactly what causes this difference. The authors of the study found that the density treatment negatively correlated with mosquito size and so reasoned that the dissemination rate could be related to smaller mosquitoes being better vectors [49]. However, another study showed that larval competition between *Ae. albopictus* and *Ochlerotatus triseriatus* led to surviving *Oc. triseriatus* mosquitoes being larger and more likely to develop disseminated infection with La Crosse virus [50]. This shows that both interspecies and intraspecies competition might ultimately affect vector competence and that this effect might not be entirely related to size.

### 3.2. Mosquito Factors

It has been apparent for many years that some mosquito species are unlikely to transmit certain viruses due to a midgut escape barrier preventing dissemination [28,33,51]. Many studies have investigated what mosquito factors contribute to the existence of this barrier, but it is often difficult to separate mosquito factors from viral factors because it has been shown that the interaction of the genotypes is important [9]. Nonetheless, several aspects of mosquito physiology have been implicated in contributing to a midgut escape barrier. These include physical characteristics of the mosquito such as basal lamina structure as well as mosquito behavior and expression of genes involved in immunity or other physiological processes.

- Physical characteristics—basal lamina thickness and structure

The basal lamina is a tightly woven extracellular matrix secreted by epithelial cells that surrounds the mosquito midgut and represents a major obstacle to disseminated infection. For years, it has perplexed researchers how viruses manage to pass through this matrix when the measured pore sizes are smaller than the size of most viruses [14]. Differences in basal lamina structure and thickness have been proposed to contribute to differences in midgut escape rates. One study found that nutritional differences led to mosquitoes of different sizes and different basal lamina thicknesses [52]. The authors found greater dissemination rates of La Crosse virus in the smaller mosquitoes that had thinner basal laminas and reasoned that this difference may, in part, explain why some mosquitoes are better vectors. However, other studies have found no association between the thickness of the basal lamina and midgut escape. A study that looked at DENV dissemination in laboratory strains of *Ae. albopictus* with differences in basal lamina thickness found no impact on viral midgut escape [53]. Other studies have found that after blood feeding, perforations appear in the basal lamina that may facilitate midgut escape [20,54]. It remains to be seen whether differences in susceptibility to these perforations is a factor in variability in midgut escape.

- Immune gene expression

(i). RNAi pathway

The RNAi pathway was first discovered in *C. elegans*, where it was unexpectedly found that double-stranded RNA could lead to the destruction or translational repression of mRNA with sequence complementarity [55]. This was subsequently demonstrated to exist in insects and to be a major contributor to the antiviral response in mosquitoes, including in the midgut [56,57,58]. Studies have provided compelling evidence that this pathway can play a major role in whether a mosquito will develop disseminated infection. For example, it has been shown that when the RNAi response was reduced in *Ae. aegypti* midgut by knocking down expression of important RNAi pathway components specifically in the midgut, there was more dissemination of SINV [59]. Conversely, when mosquitoes were genetically engineered to express inverted repeat RNA derived from DENV-2 in the midgut, which triggered an increased midgut RNAi response, there was decreased disseminated infection [60]. It should be noted that this latter study found less detectable midgut replication when the RNAi pathway was altered, which could be considered a midgut infection barrier; however, since the experiment was not designed to block entry into the midgut, but rather to suppress viral replication to levels that make midgut escape unlikely, it is being considered in our discussion here. While these studies provide compelling evidence of the importance of RNAi, we need to know if there is natural variation in the expression or functionality of RNAi pathway components, and if this can explain why some mosquitoes naturally develop disseminated infection and others do not. Evidence shows that components of RNAi like Dicer-2 can vary in their expression between different strains of mosquito species and that this differential expression may have an impact on the percentage of mosquitoes developing disseminated infection [61,62]. Exactly how much variation in the RNAi pathway contributes to midgut escape and vector competence is an area that requires more study.

(ii). Jak/STAT, Toll and IMD pathways

The antiviral activity in the midgut of mosquitoes is not limited to RNAi, as several other immune pathways have also been shown to have antiviral effects and could potentially impact midgut escape. These include the Jak/Stat, Toll and IMD pathways.

The Jak/Stat pathway has been shown to have a role in innate antiviral immunity in *Drosophila* and in mosquitoes [63,64]. When mosquitoes were engineered to have increased Jak/Stat signaling via overexpression of Dome and Hop in the fat body and midgut, a lower prevalence of disseminated DENV2 infection was observed but the prevalence of midgut infection was not altered [65]. The role of differential expression of Jak/Stat pathway components among mosquito populations with variation in midgut escape rates remains to be seen. However, expression of genes in this pathway have been found to be increased in mosquito strains that are both susceptible and refractory to DENV, which may suggest that this pathway alone cannot explain the midgut escape barrier [66].

The Toll pathway is another immune signaling cascade that has been shown to be important in innate immune defense against a variety of pathogens, including gram-positive bacteria and fungi [67]. It was subsequently shown to play a role in antiviral defense in mosquitoes [68,69,70]. There is some evidence to suggest that the basal level of activation of this pathway may vary between strains of *Ae. aegypti* as it has been found that relative REL1 expression is different in whole body samples of field-derived mosquitoes versus lab-strain mosquitoes and that these mosquito populations differ in their disseminated infection rates with DENV [62]. An additional immune pathway that has been shown to have an antiviral role in *Drosophila* is the IMD pathway [71]. This pathway has also been shown to be altered in the midgut of virally infected mosquitoes [70]. How alterations in these pathways specifically relate to midgut escape should be considered in the future.

(iii). Apoptosis and cell turnover

Apoptosis is a form of programmed cell death that is highly conserved in animals and has been extensively studied in model organisms such as *C. elegans*, *Drosophila* and mice [72]. Apoptosis is important in development and tissue maintenance, and disruptions in the process can lead to various disease states [73,74]. Importantly for the present topic, this pathway has also been known to have an antiviral role for many years [75,76]. The core mechanisms of apoptosis appear similar in many organisms. One family of important actors in the apoptotic pathway are the caspases, which are proteases that contain cysteine in their active site and are generated in an inactive form called procaspases. In response to activating stimuli, adaptor proteins bind to initiator procaspases which causes aggregation and cleavage at aspartic acid residues, resulting in vastly increased protease activity. The cleaved initiator caspases, in turn, activate effector caspases which cleave cellular targets and ultimately bring about cell death [77]. This process is highly regulated by different proteins, one of the more important families being the IAP or inhibitor of apoptosis family that was first discovered in baculoviruses [78]; subsequently, IAP homologs have been found in many organisms including mammals, *C. elegans*, insects and others [79,80,81,82]. IAP proteins bind to procaspases to prevent activation and/or bind to activated caspases to prevent their action. Many IAPs also serve as ubiquitin ligases for caspases and other targets. Another group of proteins called IAP antagonists work to prevent the action of the IAPs, which leads to caspase activation and cell death. In *Drosophila*, the genes *reaper*, *grim*, *sickle* and *hid* encode IAP antagonists. While these proteins are diverse, they all encode an N- terminal IBM or IAP binding motif which competes for caspase binding [83].

Efforts undertaken to better understand the apoptotic pathway in *Ae. aegypti* have revealed that the core pathway bears overall resemblance to the pathway in *Drosophila melanogaster*. Annotation of the *Ae. aegypti* genome has identified many homologs of known apoptosis-related genes in *Drosophila* [82,84]. The most important effector caspases in apoptosis in *Ae. aegypti* appear to be CASPS7 and CASPS8, which are homologous to DrICE and Dcp1 in *Drosophila*. These effector caspases are activated by the initiator caspase AeDronc which, in turn, is activated by the adaptor protein AeArk [85]. AeIAP1 prevents caspase activation, and silencing of this gene leads to spontaneous apoptosis in mosquito cells and mosquitoes [85,86]. The *Ae. aegypti* genome also encodes the IAP antagonists Michelob_x and IMP, which have pro-apoptotic functions [82,87,88].

Apoptosis is known to be an antiviral pathway which has implications for vector competence in mosquitoes. However, it has also been hypothesized that excess cell death in the midgut may create an opening through which viruses may escape. Some evidence suggests that midgut apoptosis varies among mosquitoes with differing levels of midgut escape. For example, midguts of a WNV-refractory *C. pipiens pipiens* strain showed evidence of apoptosis [89]. In addition, expression of caspase genes and other genes critical to apoptosis have been shown to be increased in *Ae. aegypti* mosquito strains that are refractory to DENV or in mosquito strains that show different degrees of a midgut escape barrier [90,91]. Experiments in manipulating the process of apoptosis have also suggested that this pathway may affect midgut escape. For example, when SINV was engineered to express the IAP antagonist Reaper protein, the virus rapidly lost the inserted gene after infection of *Ae. aegypti*, which suggests that expression of the proapoptotic gene was severely detrimental to virus replication [92]. In addition, in a recent study we inserted *reaper* into the SINV genome in a way that was designed to enhance stability of the insert, which resulted in significantly fewer mosquitoes developing midgut and disseminated infection when fed with this virus compared with controls [93]. However, not all evidence suggests that apoptosis is detrimental to viral dissemination. A study which tested the effect of reducing apoptosis by using RNAi to knock down expression of the gene *Aedronc* found that SINV dissemination was actually reduced [94]. Interestingly, one group has hypothesized that these apparently contradictory results may be explained by the role that *Aedronc* plays in autophagy, which may support viral replication [95]. An active area of research is how the balance of apoptosis and cell generation in the midgut affects midgut escape. A recent study found that DENV-susceptible *Ae. aegypti* mosquitoes had slower generation of new cells in the midgut [96]. This study, however, only looked at infection in the midgut and did not study disseminated infection, so this would need to be studied further to determine a specific link to midgut escape.

- Behavior—feeding behavior

A factor which is only starting to be considered as a piece in the puzzle of midgut escape is the role of mosquito feeding behavior. While on the surface it may seem that the two are unrelated, recent evidence suggests otherwise. In laboratory studies of vector competence, mosquitoes are often given a single infectious blood meal. However, this does not reflect the natural behavior of mosquitoes, since mosquitoes will often take multiple blood meals during a single gonotrophic cycle. In one study, 61% of *Ae. aegypti* mosquitoes in the lab imbibed a second blood meal, often within 24 h, and 50% of wild-caught mosquitoes showed evidence of multiple blood meals [97]. Recently, researchers have presented evidence that this behavior may improve viral dissemination from the midgut. Armstrong et al. found that when *Ae. aegypti* mosquitos received an infectious blood meal containing ZIKV and then a subsequent non-infectious blood meal, the number of mosquitoes developing a disseminated infection increased [54]. The same study reported similar results for *Ae. aegypti* and DENV, *Ae. aegypti* and CHIKV, and *Ae. albopictus* and ZIKV. Using a similar feeding regime, Kantor et al. examined the midgut after a second non-infectious feeding and found that after this blood meal CHIKV virions could be found outside of the midgut and could be seen on the basal lamina on the side of the hemocoel, while in mosquitoes fed with only a single infectious blood meal, virions were only seen in the strands of the basal lamina [20]. Another study showed that DENV4 was found in increased amounts at the midgut basal lamina of *Ae. aegypti* after a second blood meal was given, possibly impacting the likelihood of viral dissemination [21]. In the future, other aspects of mosquito behavior should be investigated in relation to midgut escape, including volume of the blood meal and number of blood meals imbibed.

### 3.3. Viral Factors

- Midgut replication

The exact role of virus replication in midgut escape has been debated and it is still unclear as to whether midgut replication is necessary in all cases, or if an extracellular (not requiring virus replication in midgut cells) dissemination route exists in some situations. Several early studies documented the appearance of viruses in the hemolymph at timepoints before they could have had time to replicate [98,99]. This led to the hypothesis that viruses may be able to move between the cells of the midgut. Further evidence for this came from a study in which the red blood cells from a blood meal were found in the hemocoel of some mosquitoes after feeding [100]. Also supporting this idea are experiments in which nanoparticles of similar sizes to arboviruses were fed to mosquitoes and were later found to have exited the midgut [21,101]. These lines of evidence suggest that midgut replication may not be required for dissemination in all cases. However, several other studies have concluded that replication is necessary for efficient midgut escape.

Studies in which the RNAi pathway in the midgut was manipulated in order to enhance or reduce virus replication showed that there was a corresponding decrease or increase in disseminated infection, indicating that the degree of virus replication influenced midgut escape [59,60]. Additionally, when GFP-expressing VEEV replicon particles that were only capable of a single round of infection were used to orally infect mosquitoes, it was found that GFP expression was limited to cells in the midgut [19]. Our recent study, in which we used a SINV construct that specifically had a reduced ability to replicate in the midgut, showed a significant reduction in the percentage of *Ae. aegypti* mosquitoes that developed disseminated infection compared with control viruses [102]. Interestingly, there was a small percentage of mosquitoes that did develop disseminated infection with this construct, but it will require more investigation to determine whether a rarely used intercellular route may exist.

Some researchers have hypothesized that a virus may need to reach a threshold level to escape from the midgut and several studies (described below) have provided evidence in support of this. Studies done with Western equine encephalitis virus (WEEV) in *Culex* mosquitoes and ZIKV in European *Ae. albopictus* mosquitoes concluded that a certain midgut level must be achieved for escape to occur [34,103]. However, other studies have refuted this idea, including one using DENV in *Ae. aegypti* [104,105]. Our recent study also did not find evidence of correlation between a high midgut titer and high carcass titer, nor did we find that midgut titer was a particularly good predictor of disseminated infection [102]. Overall, research has shown that virus midgut replication level can be an important component of midgut escape in some situations, but it may play less of a role or no role in other cases.

- Viral diversity and replication error rate

It has been well documented that the midgut represents a significant bottleneck to arbovirus genetic diversity [106,107,108]. One study estimated that in mosquitoes fed with a high dose of VEEV, the number of viruses infecting the midgut was on average around 1200, while the number of viruses escaping the midgut was only around 50 [107]. These studies bring up an important question: is having high viral diversity an advantage in overcoming the midgut escape bottleneck? Almost all arboviruses are RNA viruses with high mutation rates [109]. Researchers have been interested in determining whether decreasing the mutation rate leads to less diversity and thus less ability to overcome the challenges of midgut escape, and conversely, if there is an advantage, to increasing mutation rate. One study showed that a polymerase mutation which increased the fidelity of CHIKV replication led to decreased titers in disseminated tissues but a similar number of mosquitoes developed disseminated infection when compared with wild type infection [110]. One possible conclusion that can be drawn from this study is that decreased diversity may have led to a decrease in the number of virions able to disseminate from the midgut. Another study using high fidelity replication mutants of VEEV found significantly decreased dissemination rates [111]. Interestingly, the same study found that low fidelity mutations, which increased the mutation rate and viral diversity, also decreased the dissemination rate [111]. This can possibly be attributed to the increased accumulation of detrimental mutations. The takeaway message from these studies is that the viral RNA polymerase error rate has likely already been optimized through evolution to maximize virus success.

Studies conducted without the use of mutator variants have also implicated the importance of viral genetic diversity in dissemination. For example, one study found that SLEV that had been serially passaged in C6/36 cells displayed reduced genetic diversity compared with unpassaged virus, and when the passaged virus was fed to mosquitoes, there was a reduction in disseminated infection [112]. Taken together, these studies suggest that changes which affect viral diversity within the midgut may alter the number or ability of virions that escape the midgut.

- Co-infection

In nature, some mosquito species often populate areas where several or many different disease-causing arboviruses, parasites, and bacteria also circulate. In mosquitoes that are co-infected with a combination of pathogens, there is a need to know how these complex interactions affect midgut dissemination.

Filarial worms can cause serious disease in humans and animals, and like arboviruses, they require an insect vector to complete their life cycle. These nematodes circulate in parts of Asia, Africa and South America [113] which may also host endemic arboviruses. Research with a number of viruses has shown that mosquito ingestion of microfilariae can enhance arboviral dissemination from the midgut [114,115,116]. The reason for this is thought to be that the microfilariae puncture holes in the mosquito midgut, allowing more rapid and enhanced escape into the hemocoel. This is supported by a study that found that dissemination rates of CHIKV were increased in mosquitoes that were co-infected with *Dinofilaria immitis* microfilariae, and this correlated with holes in the midgut epithelium produced by the microfilariae [115]. Recently, it has been found that viral dissemination may not be enhanced by simply escaping through these holes but rather that viruses may be transported across the midgut epithelium by the microfilariae. When *Brugi malayi* microfilariae were incubated with EEEV or VEEV and then extensively washed and used to infect mosquitoes, many mosquitoes still became infected with the viruses [117]. This suggests that the viruses may attach to or in some other way be transported by the microfilariae. The concern is that this may lead to more hosts with complicated infections with both parasites and viruses and that ignoring the issue of parasites may compromise efforts to eliminate arboviral disease.

While co-infection with filarial worms increased arbovirus dissemination, co-infection with other arboviruses seems to have a neutral or negative effect on dissemination. Concurrent exposure of *Ae. aegypti* mosquitoes to varying combinations of CHIKV, ZIKV and DENV-2 resulted in little difference in dissemination compared with singly infected mosquitoes [118,119]. Similarly, sequential exposure to CHIKV and ZIKV did not affect dissemination rates, although transmission rates were increased [120]. There do appear to be instances of arbovirus co-infections having a negative impact on dissemination, as SINV was found to lower infection and dissemination rates of DENV-4 in *Ae. albopictus* [121]. Interestingly, mosquito infection with insect-specific flaviviruses may also have a negative effect on virus dissemination. Cell fusing agent virus (CFAV) was found to reduce dissemination rate and dissemination titer of DENV-1 and dissemination titer of ZIKV in *Ae. aegypti* [122]. Furthermore, Culex flavivirus (CxFV) was also found to affect dissemination of WNV at 7 days post-infection; however, this difference dissipated by 14 days [123]. Viral co-infection, particularly with insect-specific viruses, will be important to better understand in the future.

- Virus Dose

Available evidence suggests that midgut escape barriers can sometimes be affected by viral dose. A dose-dependent barrier can be overcome by increasing the dose of the virus to a level that may or may not be possible to attain in natural settings. Rather than some fundamental incompatibility between the virus and vector, a dose-dependent barrier may be due to a factor such as the mosquito immune response, which may be overwhelmed by a larger dose of virus. Khoo et al. supported this idea in a study which implicated the RNAi pathway in contributing to a SINV dose-dependent midgut escape barrier in *Ae. aegypti* [59]. The ability to overcome a midgut escape barrier by simply increasing the virus dose has also been shown in WEEV and *Culex tarsalis* [34,124], ZIKV and *Ae. aegypti* [125], and CHIKV in *Ae. aegypti* [126]. Understanding if a barrier is dose-dependent and the range of viral titers a vector may encounter in a natural blood meal is important for understanding vector competence.

## 4. Conclusions

It is clear that midgut escape cannot be attributed to a single factor, but that should not discourage us from attempting to understand all that we can about this enigmatic process. A better understanding of midgut escape may lead to potential new means of preventing vector infection; for example, through genetic engineering aimed at enhancing immune pathways in critical mosquito tissues or even potentially through treating mosquitoes with insect-specific viruses. This understanding also might lead to better predictions of future arboviral outbreaks. If we know how the environment, mosquito, and virus come together to promote midgut escape, we might better understand when the next significant outbreak is likely to occur and improve our preparation.

## Figures and Tables

**Table 1:** Examples of virus–vector combinations displaying a midgut escape barrier.

Virus	Vector	Reference
La Crosse virus	*Ae. triseriatus*	[28]
Jamestown Canyon virus	*Ae. provocans*	[31]
Rift Valley fever virus	*Ae. vexans* *Ae. increpitus* *Ae. melanimon* *Cx. Antennatus* *Cx. Pipiens* *Cx. quinquefasciatus*	[29,33]
dengue virus	*Ae. aegypti*	[30,32]
Western equine encephalitis virus	*Culex tarsalis*	[34]

## Data Availability

Not applicable.
